# Defective platelet function in Niemann‐Pick disease type C1

**DOI:** 10.1002/jmd2.12148

**Published:** 2020-09-12

**Authors:** Oscar C. W. Chen, Alexandria Colaco, Lianne C. Davis, Fedir N. Kiskin, Nicole Y. Farhat, Anneliese O. Speak, David A. Smith, Lauren Morris, Emily Eden, Patricia Tynan, Grant C. Churchill, Antony Galione, Forbes D. Porter, Frances M. Platt

**Affiliations:** ^1^ Department of Pharmacology University of Oxford Oxford UK; ^2^ Division in Translational Medicine Eunice Kennedy Shriver National Institute of Child Health and Human Development, National Institutes of Health, Department of Health and Human Services Bethesda Maryland USA; ^3^ Institute of Ophthalmology—Cell Biology University College London London UK

**Keywords:** calcium (Ca^2+^), lysosome, lysosome‐related organelle, Niemann‐Pick disease type C

## Abstract

Niemann‐Pick disease type C (NPC) is a neurodegenerative lysosomal storage disorder caused by mutations in either *NPC1* (95% of cases) or *NPC2*. Reduced late endosome/lysosome calcium (Ca^2+^) levels and the accumulation of unesterified cholesterol and sphingolipids within the late endocytic system characterize this disease. We previously reported impaired lysosome‐related organelle (LRO) function in *Npc1* ^*−/−*^ Natural Killer cells; however, the potential contribution of impaired acid compartment Ca^2+^ flux and LRO function in other cell types has not been determined. Here, we investigated LRO function in NPC1 disease platelets. We found elevated numbers of circulating platelets, impaired platelet aggregation and prolonged bleeding times in a murine model of NPC1 disease. Electron microscopy revealed abnormal ultrastructure in murine platelets, consistent with that seen in a U18666A (pharmacological inhibitor of NPC1) treated megakaryocyte cell line (MEG‐01) exhibiting lipid storage and acidic compartment Ca^2+^ flux defects. Furthermore, platelets from NPC1 patients across different ages were found to cluster at the lower end of the normal range when platelet numbers were measured and had platelet volumes that were clustered at the top of the normal range. Taken together, these findings highlight the role of acid compartment Ca^2+^ flux in the function of platelet LROs.

**Synopsis**

Defects in lysosome‐related organelles, specifically in platelets, are observed in the mouse model and patients with Niemann‐Pick disease type C1.

## 1 MATERIALS AND METHODS

### 1.1 Patient samples

NPC1 patients were enrolled in a longitudinal observational study (NCT00344331) at the National Institutes of Health (Bethesda, Maryland). Participants data was excluded if on an anticoagulant or post splenectomy. The NICHD Institutional Review Board approved this study; normal ranges are from the NIH Clinical Center Department of Laboratory Medicine reference ranges. Informed consent was obtained for all subjects as well as assent, when appropriate. The diagnosis was established by biochemical testing/mutation analysis.

### 1.2 Animals

Niemann‐Pick disease type C1 mice (BALB/cNctr‐*Npc1* ^*m1N*^/J, *Npc1* ^−/−^ mice) are null for Npc1 and were from an established colony. All mice were bred under sterile conditions, with food and water available ad lib. Pups were weaned 3 weeks *post‐partum*. All animal studies were conducted using protocols approved by the UK Home Office for the conduct of regulated procedures under license (Animal scientific Procedures Act, 1986). *Npc1* ^−/−^ mice have a lifespan of 10 to 12 weeks (average 10.5 weeks) with neurological symptoms presenting from 5 to 6 weeks of age.

### 1.3 Mice hematological analysis

Blood samples were obtained by cardiac puncture using an EDTA‐rinsed needle/syringe and collected into EDTA‐rinsed tubes. Multiple hematological parameters were determined using an automated blood analyzer (ABX Pentro 60 system, HORIBA‐ABX, UK).

### 1.4 Isolation of murine platelets

Platelets were harvested at indicated time points of *Npc1* ^*−/−*^ and age‐matched control littermates. For preparation of washed platelets, mice were anaesthetized and blood was collected by cardiac puncture. A total of 500 μL of blood per mouse was collected in a tube containing 100 μL acid citrate‐dextrose solution (Sigma, UK) and mixed with 3 mL 100 mM EGTA (pH 6.8) in a modified Tyrode's Ca^2+^ free buffer (134 mM NaCl, 3 mM KCl, 5 mM HEPES, 5 mM glucose, 2 mM MgCl_2_, pH 7.4) supplemented with NaHCO_3_ and BSA. Platelet‐rich plasma (PRP) was obtained by centrifugation at 180*g* for 10 minutes at room temperature. For preparation of washed platelets, PRP was washed at 1000 *g* for 8 minutes at room temperature, and the pellet was resuspended in the modified Tyrodes buffer containing prostaglandin E1 (0.25 μmol/L). Isolated mouse platelets were washed twice and either fixed for EM analysis or incubated at 37°C for 30 minutes before use.

### 1.5 Bleeding time assay

Mice were anaesthetized with isoflurane and placed onto a heated mat (37°C). The tail bleeding time was determined by removing 3 mm of the distal tail tip and immersing the tail into sterile DPBS solution (37°C). The time to cessation of bleeding was measured.^1^

### 1.6 Platelet aggregation

Platelet‐rich plasma (PRP) was prepared by centrifugation at 250*g* for 10 minutes at room temperature. The platelet count was adjusted with autologous plasma. Aggregation from PRP platelets was monitored by assessing light transmission in a microplate reader (BMG Labtech, UK) with continuous stirring at 1100 rpm at 37°C.

### 1.7 Cell culture

The human megakaryoblastic leukemia cell line MEG‐01 was obtained from the ATCC and cultured in RPMI‐1640 supplemented with 10% (vol/vol) fetal bovine serum, 1% penicillin/ streptomycin, and 0.3 μg/mL glutamine in a humidified atmosphere at 37°C and 5% CO_2_.^2^

### 1.8 Electron microscopy

DMSO or U18666A treated MEG‐01 cells were prepared for electron microscopy as previously described.^2^ Murine isolated platelets were fixed in 2% paraformaldehyde and 2% glutaraldehyde in 0.1 M sodium cacodylate for 1 hour. The cells were pelleted at 1000 g for 10 minutes and washed three times in 0.1 M sodium cacodylate prior to incubation with 1% osmium tetroxide/1.5% potassium ferricyanide on ice for 1 hour. Cell pellets were washed three times in 0.1 M sodium cacodylate, stained with 1% tannic acid for 45 minutes, dehydrated in increasing concentration of ethanol and embedded in TAAB‐812 resin. Ultrathin sections stained with lead citrate were viewed on a JOEL 1400+ transmission electron microscope (JOEL, Tokyo, Japan) equipped with a Gatan Orius SC1000B charge‐coupled device camera.

### 1.9 Lysotracker flow cytometry analysis

Washed murine platelets were diluted to 2.5 × 10^7^ platelets/mL in modified Tyrode's buffer and DMSO or U18666A treated MEG‐01 cells were resuspended in HBSS solution prior to incubation with LysoTracker Green DND‐26 for 15 minutes at room temperature.^3^ Cells were then washed with HBSS solution and flow cytometer analysis was immediately carried out.

### 1.10 Intracellular Ca^2+^ measurements

MEG‐01 cells were treated either with DMSO or 2 μM U18666A for 72 hours and then allowed to adhere to poly‐l‐lysine‐coated glass coverslips and loaded with 5 μM fura‐2/AM in the presence of 0.03% Pluronic F127 in extracellular midum (ECM, mM: 121 NaCl, 5.4 KCl, 0.8 MgCl_2_, 1.8 CaCl_2_, 6 NaHCO_3_, 25 HEPES, 10 Glucose) for 45 minutes at room temperature, followed by a 15 minutes de‐esterification. Experiments were conducted in Ca^2+^‐free medium: cells were washed once with Ca^2+^‐free ECM supplemented with 1 mM EGTA, followed by two washes in Ca^2+^‐free ECM containing 100 μM EGTA. Cells were imaged using an Olympus IX71 microscope equipped with a 40× UApo/340 objective and excited alternately by 350 nm and 380 nm light using a Cairn monochromator; emission was collected at 480 to 540 nm. Experiments were conducted at room temperature with an image collected every 3 seconds. Autoflourescence was determined at the end of each experiment by addition of 1 μM ionomycin with 4 mM MnCl_2_ to quench fura‐2. Images were analyzed using custom‐written Magipix software (Ron Jacob, King's College London, London, UK) on a single‐cell basis and the data expressed as the mean ± SEM of the maximum peak fluorescence changes (∆350/380).

### 1.11 GSL extraction, ceramide glycanase digestion, and 2AA labeling

Glycosphingolipid (GSL) extraction, ceramide glycanase digestion, and 2‐aminobenzoic acid (2‐AA) labeling were performed as described.^4^

### 1.12 Normal‐phase HPLC analysis for glycosphingolipids profile on MEG‐01 cells

Normal‐phase HPLC analysis for glycosphingolipids profiles was performed as described.^4^

### 1.13 Statistics

Data were expressed as the mean ± SEM. For group comparisons, the statistical significance of differences in mean values was determined by a two‐tailed single‐factor ANOVA, Student's *t* test or multiple *t* test using GraphPad Prism 5 for Mac OS X (version 5.0d). *P* ≤ .05 was considered significant.

## 2 INTRODUCTION

The lysosome is a regulated Ca^2+^ store and releases Ca^2+^ in response to the potent second‐messenger nicotinic acid adenine dinucleotide phosphate (NAADP).^5^, ^6^ The discovery of acidic Ca^2+^ store was made in specialized lysosome‐related organelles (LROs) found in sea urchin oocytes (yolk platelets/reserve granules).^6^ The finding that sea urchin oocyte LROs were regulated Ca^2+^ stores was subsequently replicated in conventional lysosomes, giving rise to the field of acidic store Ca^2+^ signaling.^7^ The biological roles of acidic store Ca^2+^ remain incompletely understood, but there is growing evidence that they are important in the homeostasis of the late endocytic system and in signaling cross‐talk with the endoplasmic reticulum (ER) at IP3 receptors.^8^, ^9^ LROs and secretary lysosomes are tissue/cell type specific organelles that share some common features with conventional lysosomes, including acidic pH.^10^ However, they exhibit unique morphological and functional properties, enabling them to mediate specialized functions that are distinct from conventional lysosomes. They play a particularly important role within multiple cell types of the hematopoietic system.

One approach to better understand the physiological functions of acidic store Ca^2+^ is to study inborn errors of metabolism in which this store is compromised. It has previously been identified that there is a lysosomal Ca^2+^ defect in the rare neurodegenerative lysosomal storage disease, Niemann‐Pick disease type C (NPC).^11^ NPC is caused by inherited defects in either *NPC1* or *NPC2*.^12^ We found reduced levels of Ca^2+^ in LE/Lys of NPC disease cells, likely the result of a Ca^2+^ store‐filling defect or enhanced Ca^2+^ leak.^6^ This results in insufficient Ca^2+^ release from late endosomes/lysosomes in response to the second messenger NAADP, blocking fusion, and leading to the secondary storage of multiple lipids in late endosomes (LE)/Lys, including cholesterol, sphingosine, sphingomyelin and multiple glycosphingolipids.^11^ However, the role of Ca^2+^ in LRO function and in NPC remains incompletely understood.

We previously reported that LRO function in Natural Killer (NK) cells was compromised in a mouse model of NPC disease, reducing the ability of these cells to kill target cells.^13^ Whether this is unique to NK cell biology or indicative of a broader defect in LRO function in NPC remains unknown. In this study, we have therefore investigated whether the acidic store Ca^2+^ defect found in NPC disease also affects LRO function more generally focusing on platelets, as they have both LROs and conventional lysosomes. Defects in platelets, including reduced aggregation responses to collagen, absent secondary response to epinephrine, and mean platelet volumes at the lower normal range limits, were previously observed in three NPC patients.^14^ These platelet abnormalities were further characterized in a zebrafish model of NPC disease,^14^ however it remains unclear if cholesterol storage or the calcium imbalance played a key role in contributing to these phenotypes. In this study, we detail functional deficits in platelets from NPC1 null mice and human data, supporting the hypothesis that platelet regulation is affected in NPC patients as a result of the acidic store Ca^2+^ defect.

## 3 RESULTS

### 3.1 Increased numbers of platelets (PLT), plateletcrit (PCT), and impaired thrombin‐stimulated platelet aggregation in Npc1^−/−^ mice

**FIGURE 1:**
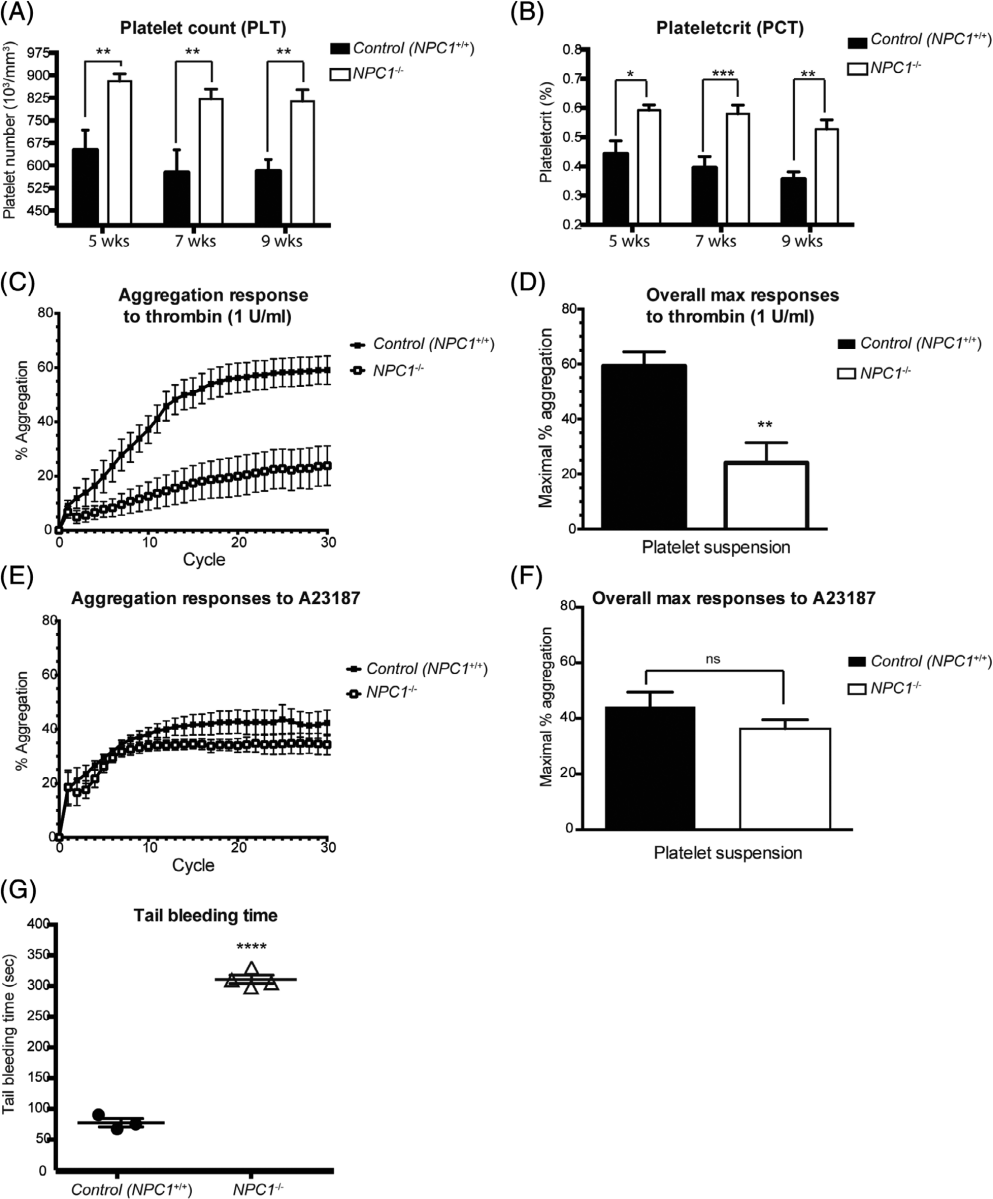
Increased platelet number (PLT), plateletcrit (PCT), impaired thrombin‐stimulated platelet aggregation and increased tail bleeding time in *Npc1* ^*−/−*^ mice. A,B, Platelet‐related parameters, including platelet number (PLT) and plateletcrit (PCT), were determined using an automatic blood analyzer. Data shown are mean ± SEM, n = 5‐7, per group. *P < .05, **P < .005, ***P < .0005, calculated by an unpaired *t* test using GraphPad Prism v5. C,D, Isolated platelets from 10‐week‐old control (*Npc1* ^*+/+*^) and *Npc1* ^*−/−*^ mice were stimulated with thrombin (1 U/mL). C, The time‐course of the aggregation response. D, Average maximal *Npc1* ^*−/−*^ mouse platelet aggregation was at 40.7% of the average maximal response relative to control mouse platelets (n = 6, per group). Data presented as mean ± SEM, *P* < .05. E,F, Isolated platelets from 10‐week‐old control (*Npc1* ^*+/+*^) and *Npc1* ^*−/−*^ mice were stimulated with the Ca^2+^ ionophore A23187 (60 μM) in the presence of CaCl_2_ (1 mM). E, The time‐course of the aggregation response. F, The maximal aggregation responses compared to the average platelet aggregation in response to A23187 (60 μM) was 44.2% for control mouse platelets and 36.2% for *Npc1* ^*−/−*^ mouse platelets, respectively. This difference was not statistically significant, n = 4‐5, per group. Data presented as mean ± SEM. G, Tail bleeding assays were performed as described on 10.5‐week‐old control (*Npc1* ^*+/+*^) and *Npc1* ^*−/−*^ mice. The time from incision to cessation of bleeding was recorded. Data represent mean ± SEM, n = 3‐4, per group

To measure hematological parameters, whole blood was collected from presymptomatic (5 weeks), early symptomatic (7 week), and late stage (9 week) *Npc1* ^−/−^ mice. There was a significant increase in the circulating platelet count (PLT) (Figure 1A) and plateletcrit (percentage volume of platelets in the blood, PCT) in *Npc1* ^−/−^ mice (Figure 1B). The number of platelets in the *Npc1* ^−/−^ mice was significantly elevated from 5 weeks of age (Figure 1A, PLT: 5 weeks, *P* = .0098; 7 week, *P* = .0026; 9 weeks, *P* = .0086), as was the plateletcrit compared to wild type controls (Figure 1B PCT: 5 weeks, *P* = .0127; 7 weeks, *P* = .0009; 9 weeks, *P* = .004). However, there was no significant change in mean platelet volume (MPV) in *Npc1* ^*−/−*^ platelets compared with control (*Npc1* ^*+/+*^) platelets at any age (data not shown). There was a significant decrease in *Npc1* ^−/−^ platelet aggregation by 40.7% in response to thrombin stimulation (1 U/mL) compared with control (*Npc1* ^*+/+*^) mouse platelets (Figure 1C,D; *P* = .0029). However, there were no significant differences in *Npc1* ^*−/−*^ platelet aggregation in response to the Ca^2+^ ionophore A23187 compared with controls (Figure 1E,F; *P* = .2714).

### 3.2 Increased bleeding time in Npc1^−/−^ mice

As *Npc1* ^*−/−*^ mice have an abnormal circulating platelet count and impaired thrombin induced‐platelet aggregation, we next investigated whether this would lead to bleeding abnormalities. A tail‐bleeding assay was performed on anaesthetized 10.5‐week‐old *Npc1* ^−/−^ mice (late‐stage disease) and we found a marked hemostatic defect in *Npc1* ^−/−^ mice (Figure 1G). Whereas bleeding spontaneously stopped after approximately 2 minutes in control age‐matched (*Npc1* ^+/+^) mice, tail‐bleeding time was markedly increased in 10.5‐week‐old *Npc1* ^−/−^ mice (Figure 1G, *P* < .0001).

### 3.3 Abnormal ultrastructure in Npc1^−/−^ platelets

**FIGURE 2:**
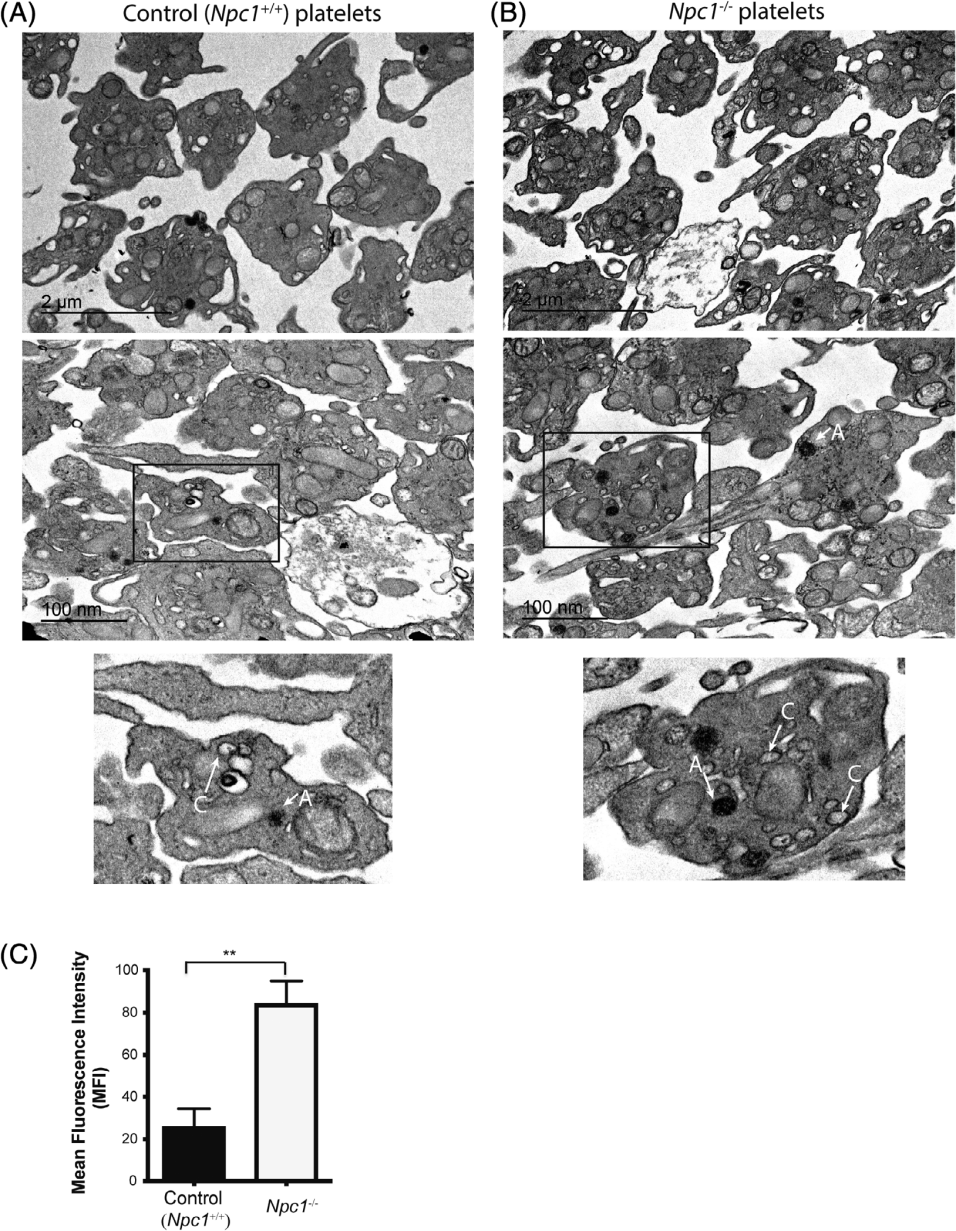
Abnormal platelet lysosomal morphology in the *Npc1* ^*−/−*^ mice. Representative images of transmission electron microscopy analysis from, A, control (*Npc1* ^+/+^) and B, *Npc1* ^−/−^ murine platelets. Arrows indicate alpha granules (A) and cytoplasmic vacuoles (C). C, Significant enlargement of the lysosomal/acidic compartment volume using lysotracker staining in *Npc1* ^−/−^ murine platelets was observed compared to control *Npc1* ^+/+^ platelets. Data were quantified by flow cytometry from three independent experiments. *P < .05. Data are presented as mean ± SEM, n = 3, calculated by student *t* test and plotted using GraphPad Prism v5

To determine whether there were any ultrastructural changes in *Npc1* ^−/−^ platelets we analyzed them by transmission electron microscopy. Compared to control wild type (*Npc1* ^+/+^) platelets (Figure 2A), numerous abnormal and heterogeneous electron‐dense structures resembling lysosomal storage bodies as well as increased cytosolic vacuoles were observed in the *Npc1* ^−/−^ platelets (Figure 2B, alpha granules (A) and cytosolic vacuoles (C) indicated in inset). In general, the electron density of the *Npc1* ^−/−^ platelets was also higher than in control platelets consistent with lipid storage. In line with the EM images, the volume of the isolated platelet acidic organelles was measured using LysoTracker. There was a significant increase in acidic organelle volume in the *Npc1* ^−/−^ platelets as compared to the wild type *Npc1* ^+/+^ platelets (*P* = .0025, Figure 2C).

### 3.4 Increased LysoTracker fluorescence, presence of storage bodies, and elevated GSLs in U18666A‐treated human megakaryoblastic MEG‐01 cell line

**FIGURE 3:**
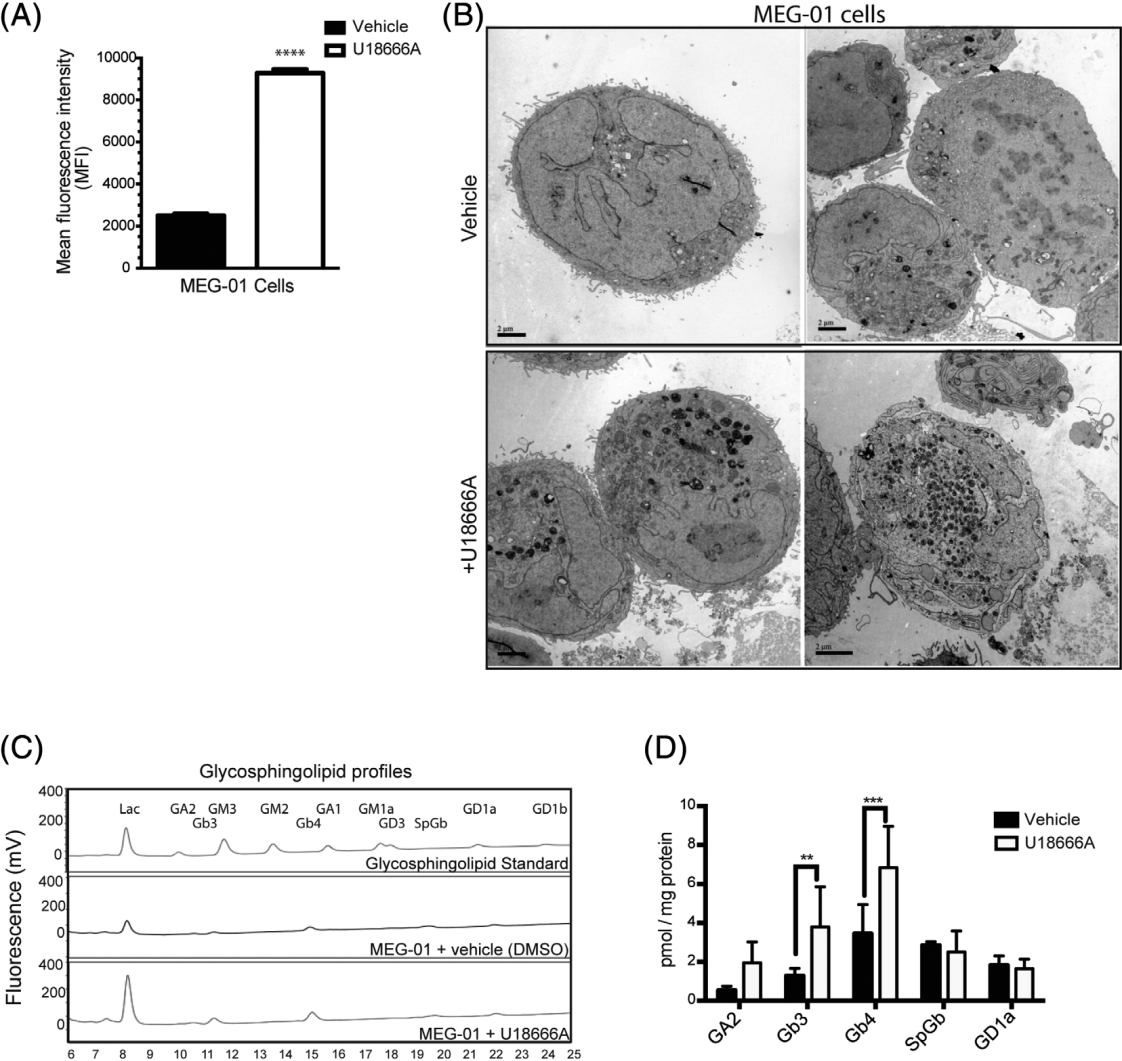
Enlarged acidic compartments are present and accumulates GSLs in U18666A‐treated MEG‐01 cell line. A, Quantitative data of flow cytometric analysis of the numbers/volume changes of acidic compartments in vehicle (DMSO) or U18666A‐treated MEG‐01 cells using lysotracker staining. Data were quantified from three independent experiments. ****P < .00005. Data are presented as mean ± SEM, n = 3, calculated by student *t* test and plotted using GraphPad Prism v5. B, Representative images of transmission electron microscopy analysis from vehicle (DMSO) and U18666A‐treated human megakaryoblastic MEG‐01 cell lines. Scale bar = 2 μm. C, HPLC analysis of GSLs compositions of DMSO, U18666A treated MEG‐01 cells. Profiles are representative of three independent analyses. D, Bar graph comparing GSLs contents in DMSO, U18666A treated MEG‐01 cells. MEG‐01 cells were treated either with DMSO or U18666A for 72 hours as described in Section 1. Data shown are mean ± SEM, n = 3, per group. *P ≤ .05, **P ≤ .01, ***P ≤ .001, calculated by one‐way ANOVA with multiple comparisons using GraphPad Prism v5

To further investigate the underlying mechanisms of *Npc1* ^*−/−*^ platelet dysfunction, we used the pharmacological agent U18666A (2 μM) to induce NPC1 disease‐associated cellular phenotypes in the human megakaryocyte line MEG‐01, as these cells are responsible for the production of platelets. U18666A is an inhibitor of NPC1 and has recently been shown to directly bind to the NPC1 protein.^15^ The volume of acidic organelles in MEG‐01 cells were measured and there was a significant increase in LysoTracker‐Green signal intensity in U18666A‐treated MEG‐01 cells compared with vehicle‐treated (DMSO) cells (*P* < .0001) (Figure 3A). Transmission electron microscopy analysis revealed the presence of numerous enlarged electron dense storage bodies in U18666A‐treated MEG‐01 cells (Figure 3B) suggesting that U18666A‐treated MEG‐01 cells could act as a model for studying phenotypes relevant to mouse platelets. Furthermore, U18666A or vehicle‐treated MEG‐01 cellular glycosphingolipid (GSL) levels were measured by HPLC analysis and we found significantly elevated levels of Gb3 and Gb4 in U18666A‐treated MEG‐01 cells, compared with vehicle treated cells (Figure 3C,D, Gb3 *P* = .0074, Gb4 *P* = .0004).

### 3.5 Defects in acidic compartment Ca^2+^ flux in U18666A‐treated MEG‐01 cell line

**FIGURE 4:**
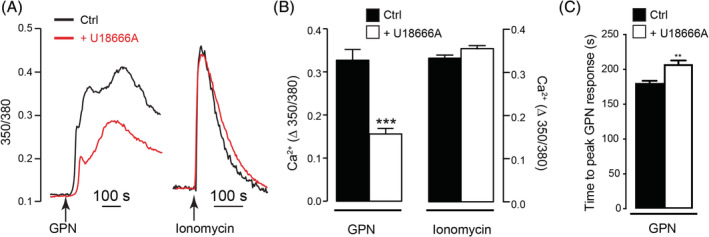
Defects in acidic compartment Ca^2+^ flux in U18666A‐treated MEG‐01 cell line. A, Representative single‐cell Ca^2+^ traces showing 350/380 ratios of fura‐2 fluorescence and B, maximum Ca^2+^ changes upon addition of 200 μM GPN or 1 μM ionomycin in MEG‐01 cells with or without 72 hours pretreatment with 2 μM U18666A. DMSO was used as a vehicle control and no Ca^2+^ release was observed (not shown). C, Time‐to‐maximum response upon GPN addition following DMSO (Ctrl) or U18666A pretreatment in MEG‐01 cells. n = 383 (GPN Ctrl), 450 (GPN U18666A), 285 (Iono Ctrl), 279 (Iono U18666A) cells. Data are represented as mean ± SEM and statistical significance was determined using an unpaired 2‐tailed *t* test. **P < .01. ****P < .0001, ns *P* > .05

To assess the effect of U18666A treatment on lysosomal Ca^2+^, we monitored Ca^2+^ content in MEG‐01 cells indirectly by releasing Ca^2+^ from the lysosome lumen to the cytosol with the lysomotropic agent glycyl‐l‐phenylalamine 2‐napthylamide (GPN).^16^ All experiments were performed in Ca^2+^‐free medium to eliminate Ca^2+^ influx. We have previously shown that GPN responses faithfully reflect lysosomal Ca^2+^ levels in NPC1 patients‐derived human fibroblasts and U18666A‐treated RAW cells.^11^ In agreement with known NPC cellular phenotypes,^11^, ^13^ U18666A‐treated MEG‐01 cells exhibited a significant decrease in GPN‐stimulated Ca^2+^ release compared to vehicle (DMSO)‐treated cells (Figure 4A,B, *P* < .0001), consistent with less Ca^2+^ within the lysosomes of U18666A‐treated MEG‐01 cells. The major effect of U18666A on the GPN response is on the maximum amplitude of the Ca^2+^ response (48% reduction), whilst there is only a small (13%) difference in the kinetics (time from GPN addition to the maximum amplitude) (Figure 4C, *P* = .0014).

Because GPN responses can be a summation of Ca^2+^ release from both the lysosome and the endoplasmic reticulum (ER),^16^ the reduced GPN response could also reflect a lower ER Ca^2+^ content. To discount this explanation, the ER content was assessed with the Ca^2+^ ionophore, ionomycin. Ionomycin was used rather than the SERCA inhibitor, thapsigargin, because it is a more reliable assessment of the ER content: thapsigargin releases ER Ca^2+^ so slowly that other Ca^2+^ removal processes dampen both its kinetics and amplitude,^17^, ^18^, ^19^, ^20^ and whether NPC alters these removal processes is unclear. By contrast, ionomycin releases ER Ca^2+^ with such rapid kinetics that it is less susceptible to Ca^2+^ buffering processes. In contrast to GPN‐dependent Ca^2+^ release, ionomycin‐dependent Ca^2+^ release from the ER was unaffected by U18666A treatment (Figure 4A,B, *P* = .1643). Together, these data are consistent with U18666A‐treatment reducing the lysosome (but not ER) Ca^2+^ content in MEG‐01 cells.

### 3.6 NPC1 patient platelet counts and volumes cluster at the extremes of the normal ranges

**FIGURE 5:**
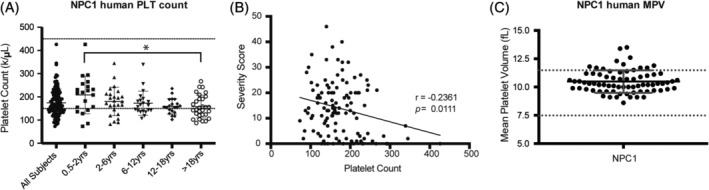
NPC1 patients present at the extremes of normal hematological ranges. A, Platelet count (PLT) was determined using an automated blood analyzer. Dashed lines indicate the estimated normal range (150‐450 k/μL). Patients over 18 years of age had significantly reduced platelet counts as compared to patients age 0.5 to 2 years. n = 116. B, NPC1 patient severity and platelet counts negatively correlate as calculated by Pearson *r*‐coefficient. Severity scores calculated by scoring nine major domains (ambulation, cognition, eye movement, fine motor, hearing, memory, seizures, speech, and swallowing) and eight minor domains (auditory brainstem response, behavior, gelastic cataplexy, hyperreflexia, incontinence, narcolepsy, psychiatric, and respiratory problems).^22^ C, NPC1 patient mean platelet volume (MPV) was determined using an automated blood analyzer. n = 73, dashed lines indicate normal range: 7.5‐11.5. Data shown are mean ± SEM, statistical significance calculated by a one‐way ANOVA, Tukey's multiple comparisons test using GraphPad Prism v5. *P < .05

Platelet counts were measured in NPC1 patients. As illustrated in Figure 5A, NPC1 patients had mean platelet count values of approximately 174 (k/μL) clustered at the bottom of the normal range (normal range: 150‐450 k/μL). Additionally, in line with previous studies,^21^ the platelet counts decrease in age with the patients >18 years have significantly less platelets than the 0.5 to 2 year old patients (*P* = .0345). Platelet counts in the NPC1 patients also significantly correlate with severity score,^22^ with more severe patients having lower platelet counts then less severe patients (Pearson's correlation coefficient *r* = −.2361, *P* = .0111, Figure 5B). When platelet volumes were measured the mean platelet volumes were towards the upper limit (mean value: 10.48 fL) of the normal range (normal range: 7.5‐11.5 fL) (Figure 5C).

## 4 DISCUSSION

We have found an elevated number of circulating platelets, defective platelet aggregation, abnormal platelet morphology and prolonged bleeding times in *Npc1* ^*−/−*^ disease mice. Electron microscopy revealed abnormal ultrastructure consistent with lipid storage in *Npc1* ^*−/−*^ platelets and in the U18666A‐treated MEG‐01 cell line. Furthermore, U18666A‐treated MEG‐01 cells had reduced levels of acidic store Ca^2+^ and glycosphingolipid storage. Taken together, these data suggested that megakaryocytes/platelets have functional defects in NPC disease.

We found that *Npc1* ^*−/−*^ platelets exhibit functional defects specifically when simulated with thrombin. It has been suggested that platelet lysosome secretion is only activated by potent agonists, such as thrombin.^23^, ^24^ On the other hand, when *Npc1* ^*−/−*^ platelets were stimulated with A23187, a Ca^2+^ ionophore selectively transporting Ca^2+^ across cell membranes into the cytoplasm or releasing the divalent cation from intracellular storage sites, the *Npc1* ^*−/−*^ platelets did not exhibit aggregation defects.^25^ Thrombin activation of platelets is known to affect IP_3_‐signaling, resulting in an increased intracellular Ca^2+^ flux.^26^ Therefore, this finding suggests the agonists‐mediated aggregation defects occurs in *Npc1* ^*−/−*^ platelets due to lysosomal calcium defects.

As anticipated,^27^ platelet count levels towards the lower end of the normal range was observed in our clinical hematological studies in NPC1 disease patients; however, thrombocytosis was observed in the *Npc1* ^*−/−*^ mice. The reason for this difference is unknown and could be a species‐specific difference or reflect the fact that the mouse model is completely null for *Npc1* whereas the patients typically have some residual NPC1 expression leading to less extreme cellular phenotypes. Another possible explanation is that there could be a platelet clearance defect and altered physiological distribution of platelets in NPC1 disease, since enlargement and fibrosis of the spleen occurs in NPC1 patients and *Npc1* ^*−/−*^ mice.^28^ Platelets may be trapped in the spleen and affect circulating platelets numbers. Therefore, in vivo platelet clearance assays could be helpful to clarify these species‐specific platelet dysregulation phenotypes.

However, we still cannot exclude a potential platelet production defect due to impaired megakaryocyte function in NPC1, and in fact it has been suggested that NPC1 plays a role in the regulation of thrombocyte formation in zebrafish.^14^ Sphingosine‐1‐phosphate (S1P) and its associated receptors, especially S1P_1_ and S1P_4_ receptors, have also been suggested to be involved in the regulation of megakaryopoiesis, pro‐platelet formation and shedding in vivo and in vitro models.^29^ Furthermore, S1P_4_ receptor‐null megakaryocytes exhibited abnormal cellular morphology, which was characterized by cytoplasmic vacuolation and nuclear‐ploidy.^30^ Interestingly, abnormal platelet formation and megakaryopoiesis defects have been reported in a few clinical cases of NPC1 and it has been suggested that this could be associated with cholesterol storage in NPC1 disease.^14^ Since the platelet lipid compositions and granule contents are mainly determined during megakaryopoiesis and platelet shedding, the abnormal *Npc1* ^*−/−*^ platelet phenotypes could also be associated with abnormal intracellular lipid trafficking and storage phenotypes during megakaryopoiesis which could impair down‐stream lipid‐mediated intracellular signaling.^31^

*Npc1* ^*−/−*^ platelets functional defects could also primarily be associated with acidic compartment Ca^2+^ signaling defects. Although the physiological functions and activation mechanisms of platelet LROs remain incompletely understood, our current studies highlight the importance of acidic store Ca^2+^ signaling in the regulation of platelet function. NAADP is a novel Ca^2+^‐mobilizing second messenger, which selectively targets acidic organelles.^32^ Thrombin and collagen‐related‐peptides stimulated human platelet aggregation and activation, including fibrinogen binding and granule release, is highly dependent on NAADP‐mediated Ca^2+^ signaling, which suggest a crucial role of NAADP and acidic store Ca^2+^ release during platelet activation.^33^, ^34^ Furthermore, NPC1 patients were shown to have reduced platelet aggregation responses to low concentration of collagen and an absent secondary response to epinephrine,^14^ consistent with the agonist mediated platelet activation defects in the NPC1 mouse model. Therefore, our hypothesis is that *Npc1* ^*−/−*^ platelet functional defects are primarily associated with impaired NAADP‐mediated acidic store Ca^2+^ signaling.

Furthermore, similar observations of abnormal platelet granule morphology and platelet functional defects have been reported in other lysosomes/LROs dysfunctional diseases, such as Tangier disease, Chediak‐Higashi syndrome and Hermansky‐Pudlak syndrome.^35^, ^36^, ^37^ Very significantly Tangier disease has recently been found to share cell biological and biochemical features of NPC disease, including reduced lysosomal Ca^2+^ storage. Additionally, one patient with Tangier disease treated with miglustat showed a clinical response to this NPC therapeutic suggesting pathogenic convergence.^38^, ^39^ Collagen and thrombin induced aggregation defects were also observed in a mouse model of Tangier disease and individuals with Tangier disease, suggesting agonists‐mediated platelet activation signaling defects in Tangier disease.^37^

One of the common features of these lysosomes/LROs dysfunction disorders is that they all have intracellular Ca^2+^ homeostasis defects, either in the ER or LE/Lys Ca^2+^ stores.^11^, ^37^, ^40^ Since intracellular Ca^2+^‐mediated signaling is crucial for intracellular vesicle fusion and fission events, acidic compartment‐mediated Ca^2+^ homeostasis defects could lead to abnormal intracellular vesicle trafficking and storage in these diseases. Therefore, it would be reasonable to hypothesize that acidic stores Ca^2+^ homeostasis defects in NPC1 disease could impair subcellular trafficking, affect granules content secretion and lead to the activation/aggregation defects observed in *Npc1* ^*−/−*^ megakaryocytes/platelets. Better understanding the role of calcium signaling in platelets is important, as both NPC1 and Tangier patients have decreased plasma HDL‐C cholesterol levels, which are associated with an increased risk for all forms of atherosclerotic diseases, including myocardial infarction and stroke.^41^, ^42^

Taken together, our current studies have demonstrated that *Npc1* ^*−/−*^ platelets have functional defects in a murine model of NPC1 disease. Furthermore, our studies suggest that *Npc1* ^*−/−*^ platelet functional defects could be associated with impaired acidic stores Ca^2+^ signaling in NPC1 disease model and implicates acidic store Ca^2+^ to be further examined in better understanding the homeostasis of LROs.

## CONFLICT OF INTEREST

The authors have no conflict of interest.

## INFORMED CONSENT

All procedures followed were in accordance with the ethical standards of the responsible committee on human experimentation (institutional and national) and with the Helsinki Declaration of 1975, as revised in 2000.^5^ Informed consent was obtained from all patients for being included in the study.

## ANIMAL RIGHTS

All institutional and national guidelines for the care and use of laboratory animals were followed.

## AUTHOR CONTRIBUTIONS

O.C.‐W.C., A.C., L.C.D., F.N.K., and A.O.S. developed methods, devised, and performed experiments; O.C.‐W.C., A.C., L.C.D., and F.N.K., analyzed the data and performed statistical analysis; D.A.S. and L.M. helped with animal studies; P.T. and E.E. performed the transmission electron microscopy. F.D.P. and N.Y.F. obtained ethical permission, collected clinical samples, and provided data; O.C.‐W.C., A.O.S., G.C.C., A.G., F.D.P., and F.M.P. devised and designed the research; O.C.‐W.C., A.C., and F.M.P. wrote the article.

## ETHICS APPROVAL

NPC1 patients were enrolled in a longitudinal observational study (NCT00344331) at the National Institutes of Health (Bethesda, Maryland, USA). The NICHD Institutional Review Board approved this study. All procedures followed were in accordance with the ethical standards of the responsible committee on human experimentation (institutional and national) and with the Helsinki Declaration of 1975, as revised in 2000. All animal studies were conducted to comply with the ARRIVE guidelines using protocols approved by the UK Home Office for the conduct of regulated procedures under license (Animal scientific Procedures Act, 1986).

## PATIENT CONSENT

No identifiable personal data is included in this article.

## DATA AVAILABILITY STATEMENT

Data sharing is not applicable to this article as no datasets were generated or analyzed during the current study.
